# Cellulose Fibre-Reinforced Biofoam for Structural Applications

**DOI:** 10.3390/ma10060619

**Published:** 2017-06-06

**Authors:** Jasmina Obradovic, Mikko Voutilainen, Pasi Virtanen, Lippo Lassila, Pedro Fardim

**Affiliations:** 1 Department of Fibre and Cellulose Technology, Åbo Akademi, Porthansgatan 3, 20500 Turku, Finland, jobradov@abo.fi; 2 Department of Chemistry, University of Helsinki, A.I. Virtasen Aukio 1, 00100 Helsinki, Finland, ma.voutilainen@helsinki.fi; 3 Department of Industrial Chemistry and Reaction Engineering, Åbo Akademi, Biskopsgatan 8, 20500 Turku, Finland; pasi.virtanen@abo.fi; 4 Department of Prosthetic Dentistry and Biomaterials Science, University of Turku, Itäinen Pitkäkatu 4 B, 20500 Turku, Finland; liplas@utu.fi; 5 Department of Chemical Engineering, KU Leuven, 3000 Leuven, Belgium

**Keywords:** AESO, bio-based, cellulose, foam, renewable resource

## Abstract

Traditionally, polymers and macromolecular components used in the foam industry are mostly derived from petroleum. The current transition to a bio-economy creates demand for the use of more renewable feedstocks. Soybean oil is a vegetable oil, composed mainly of triglycerides, that is suitable material for foam production. In this study, acrylated epoxidized soybean oil and variable amounts of cellulose fibres were used in the production of bio-based foam. The developed macroporous bio-based architectures were characterised by several techniques, including porosity measurements, nanoindentation testing, scanning electron microscopy, and thermogravimetric analysis. It was found that the introduction of cellulose fibres during the foaming process was necessary to create the three-dimensional polymer foams. Using cellulose fibres has potential as a foam stabiliser because it obstructs the drainage of liquid from the film region in these gas-oil interfaces while simultaneously acting as a reinforcing agent in the polymer foam. The resulting foams possessed a porosity of approximately 56%, and the incorporation of cellulose fibres did not affect thermal behaviour. Scanning electron micrographs showed randomly oriented pores with irregular shapes and non-uniform pore size throughout the samples.

## 1. Introduction

Polymer foams are a group of lightweight, very porous, and low-density materials. Generally, foams can be classified as flexible or rigid, closed- or open-cell foam [1]. Microporous polymers are ideal for application where weight saving is critical, such as thermal insulation [2], sandwich structures [3], and packaging [4]. The majority of commercial polymeric foams are petrochemical-based.

Numerous studies have explored the possibility of developing foams from biomass [5,6,7]. The bulk of these studies have investigated the possibility of producing polyurethane foams from vegetable oils. Polyurethanes foams are produced by the reaction of a polyol with a diisocyanate. Polyurethanes from vegetable oils are based on polyfunctional polyols that form crosslinked products. The properties of such products depend on the crosslinking density of the networks and their chemical composition. Polyols can be synthesised from soybean, palm, castor, sunflower, linseed, and canola oil [8,9,10,11]. Soybean oil-based polyols can be produced via epoxidation route followed by ring opening, hydroformylation, ozonolysis, or transesterification [12]. However, current plant-based foams still contain a large fraction of fossil-based components.

Research attention has been given to thermosetting foams, because they were likely to present higher mechanical properties. Vegetable oils can be cured after epoxidation [13], acylation [14], and maleination [15]. Bonniallie and Wool reported the preparation of thermosetting foams from acrylated epoxidized soybean oil using the pressurised carbon dioxide foaming process [14]. The foams density was controlled by the CO_2_ pressure inside the reactor and by the vacuum applied during curing. The resulting foams had mechanical properties comparable with semi-rigid industrial foams.

A mechanical frothing technique for producing macroporous acrylated epoxidized soybean oil (AESO) foam has been reported by Lee and co-workers [1]. The authors utilised the mechanical frothing to create a gas-liquid foam from soybean-derived renewable monomers mixed with bacterial cellulose (BC) nanofibrils dispersed in the monomer phase. Mechanically frothed gas-AESO-BC foam was polymerised through microwave heating containing lauroyl peroxide as a thermal initiator. The resulting bio-based macroporous polymer had a porosity of 60%, and its foam stability was higher in the presence of bacterial nanofibrils. This was thought to be to the obstruction of the Plateau border in the presence of bacterial cellulose during capillary drainage of the monomer liquid. Bacterial cellulose contributed as a reinforcement to the higher mechanical properties. Wu and co-workers reported a biofoam composite prepared using short sisal fibres as a reinforcement and acrylated epoxidized soybean oil as a matrix [16]. The usage of volatile toxic styrene monomers and accelerant *N*,*N*-dimethylaniline was avoided in the current study. A study of the failure mechanism revealed that the adhesion between the fibre and the matrix was a key issue responsible for damage of the foam, therefore interfacial interactions were enhanced with the surface treatment of sisal fibres. Soil burial tests provided the information that the foams could be biodegradable.

In the present study, we report the preparation of thermosetting acrylated epoxidized soybean oil foams using different cellulose fibre content (2%, 3% and 4%) as a reinforcement component. Unlike previously published research, reported here is a simple preparation of bio-based foam without the usage of toxic chemicals. Consequence of avoiding addition of styrene was incorporation of shorter fibres. Sodium bicarbonate was selected as a low-cost, nontoxic, and thermally safe latent foaming agent, and sodium lauryl sulfate was chosen as a surfactant to stabilise the foam. The physical and mechanical properties of the bio-based foam were assessed, and the effect of the reinforcing agent content on the foams was discussed.

## 2. Materials and Methods

### 2.1. Materials

Enoalfa dissolving pulp, produced by Stora Enso Enocell pulp mill in Finland were used in foam preparation. Enoalfa dissolving pulp was produced from a mixture of aspen and birch, with 93.5% alpha cellulose content. Acrylated epoxidized soybean oil (>99%, Sigma-Aldrich, St. Louis, MO, USA), t-butyl peroxybenzoate (>99%, Sigma-Aldrich, St. Louis, MO, USA), sodium hydrogen carbonate (Merck, Branchburg, NJ, USA), and sodium dodecyl sulfate (Sigma-Aldrich, St. Louis, MO, USA) were used as received without further purification.

### 2.2. Bio-Based Foam Preparation

Enoalfa dissolving pulp was blended in a PolyMIX machine for 30 s, prior to foam formation. Pulp fibres were approximately 500 mm in length and 10 mm in diameter. Polymer foams were prepared by polymerising the gas-fibre-AESO mixture. Acrylated epoxidized soybean oil (10.0 g) was mixed with 1.0 cm^3^ t-butyl peroxybenzoate, 0.4 g anionic surfactant (CH_3_(CH_2_)_11_SO_4_Na), and 0.5 g blowing agent (NaHCO_3_) in a laboratory aluminum pan. The cellulose fibres were introduced into the mixture in different weight fractions (2.0%, 3.0% and 4.0%) in relation to the total amount of AESO monomer. The addition of cellulose fibres increased the viscosity of the mixture. The chemical blowing agent reacted when 0.5 mL of distilled water was added into the system. The mixture was then placed in an oven for 4 h at 80 °C. After polymerisation, the foams were left at ambient temperature to cool down for 24 h. The post-curing procedure was performed at 110 °C for 30 min. The final foam had a top diameter of 50 mm, a bottom diameter of 42 mm, and was 10 mm high.

### 2.3. Porosity Determination Using Argon Pycnometer

Grain volumes of the samples were measured using an Ar-gas pycnometer whose operation was based on applying the equation of the state of ideal gas. The measurement setup consisted of two chambers (sample and reference), pressure gauges and temperature gauges, an Ar-gas supply, and a vacuum pump. A more detailed description of the method and equipment is given by Voutilainen et al., 2012 [17]. The bulk volumes of the samples were determined by measuring the sample dimensions using a vernier calliper. The uncertainties of the measurements were determined using the general law for propagation of uncertainty. Porosity (*ε*) was defined using the following equation:

(1) $$\varepsilon \;=\frac{V_{B}-V_{G}}{V_{B}}\times 100\%$$

where *V*_B_ is the bulk volume and *V*_G_ is the grain volume of the sample.

The grain volume measurement results were averages over 10–12 independent measurements. The height of the sample was measured from several (4–6) locations and their average was used when determining the bulk volumes. The given value for the pristine soy bean oil sample was highly uncertain due to the isolated pores that were not taken into account using pycnometry. When using an Ar-gas pycnometer, the gas can only penetrate into pores that are connected to the surface (directly or via other pores), and thus the given value overestimates the true grain volume and underestimates the porosity. Grain density and bulk density were calculated by dividing the mass of the sample by the grain volume and bulk volume.

### 2.4. Field Emission Scanning Electron Microscope (FE-SEM)

The morphology of cellulose-reinforced AESO foams was examined by a Leo Gemini 1530 field emission scanning electron microscope with an In-Lens detector. Samples were coated with carbon in a Temcarb TB500 sputter coater (Emscope Laboratories, Ashford, UK). The optimum accelerating voltage was 2.70 kV.

### 2.5. 3D Optical Microscope

Non-invasive 3D surface measurements of cellulose-reinforced AESO foams were examined by Bruker’s 3D optical microscope system (Bruker Nano GmbH, Berlin, Germany). Bruker Vision64 software provided a functional and streamlined graphical user interface, as well as comprehensive data collection and analysis.

### 2.6. Compression Test

The compressive properties of the cellulose-reinforced AESO foams were evaluated on an Instron universal testing machine (Instron 8872, Instron Corporation, Norwood, MA, USA) with a crosshead speed of 10 mm/min. The compression force was applied in parallel to the foam rise direction. At least five samples were tested to obtain average values. The test specimens were cubic with a 10-mm long edge, except for the pristine AESO sample that was parallelepiped (10 × 10 × 5 mm^3^). Bluehill 3 software provided data collection and analysis.

### 2.7. Nanoindentation Analysis

The mechanical properties of cellulose-reinforced AESO foams were measured with a UBI1 Nanomechanical Test Instrument (HYSITRON, Inc., Minneapolis, MN, USA) using a continuous stiffness measurement in a force controlled mode with a Berkovich type triangular diamond pyramid. The continuous stiffness measurement technique gives access to contact stiffness, hardness, elastic modulus, and creep resistance. Nanoindentation reduced modulus (*E*) and hardness (*H*) are defined by the following equations:

(2) $$E=\frac{1}{2}\frac{dP}{dh}\sqrt{\frac{\pi }{A}}$$

(3) $$H=\frac{P_{max}}{A}$$

with *P_max_* is the applied load at the maximum depth of penetration, *A* is the contact area and *dP/dh* is the slope of the initial portion of the unload curve in the load-displacement plot. At least five indentations were performed on each sample, with a peak load force of 200 µN, and the average of these values was calculated. The nanoindentator was calibrated against a polycarbonate standard with a maximum standard deviation of 10%.

### 2.8. Thermal Analysis

The thermogravimetric analyses of the cellulose-reinforced AESO foams were carried out in a TGA-DTA thermoanalyser (Q Series instrument, TA instruments, DE, USA). The specimens of 80–100 mg each were heated in corundum crucibles up to 500 °C at a heating rate of 10 °C per min in argon atmosphere.

## 3. Results and Discussion

### 3.1. Structure and Porosity of Bio-Based Foams

Each foam pore is a volume of gas enclosed in polymer walls. In solid foams, a cell with all its surrounding walls intact is called a closed-cell. When at least two walls are broken during the solidification phase of the foam, the cell is called an open-cell. Polymeric foams consist of a mixture of open- and closed-cells in varied proportions [18]. The mechanical and physical properties of thermosetting foams are related to the foam structure, which is controlled by the rates of bubble nucleation, bubble growth, foam aging, and polymerisation according to the laws of kinetics, thermodynamics, and transport phenomena. In this work, we decided to fix the monomer AESO, the type of foaming process, and the blowing agent as well as to focus on the reinforcement phase and the effect of cellulose fibres on overall porous polymer properties. The pores in the foam were formed by means of the chemical blowing agent followed by thermal polymerisation of the liquid monomer. Acrylated epoxidized soybean oil is fascinating due to the high reactivity of the acrylic groups pertaining to easy polymerisation via free radical reactions.

**Figure 1:**
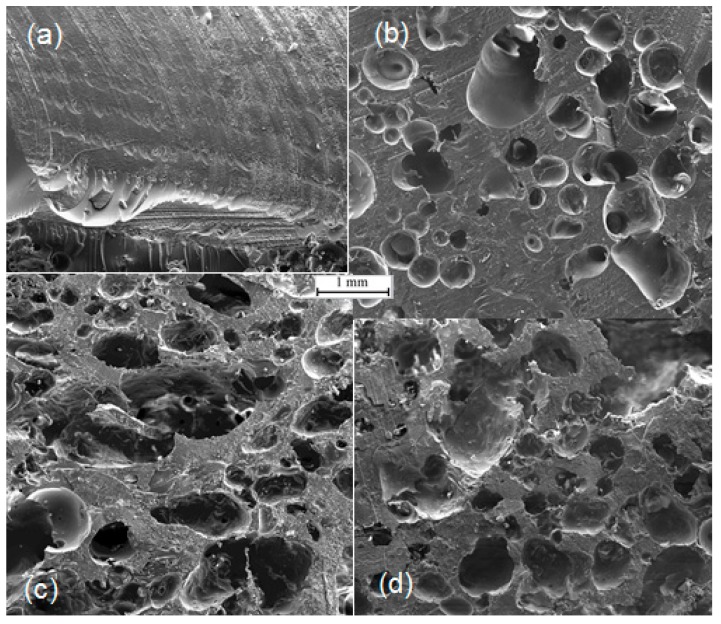
The SEM images of foams consisting of pristine AESO material (**a**); as well as AESO foam reinforced with 2.0% cellulose fibres (**b**); 3.0% cellulose fibres (**c**); and 4.0% cellulose fibres (**d**).

SEM images showing the internal structure and morphology of the pristine AESO material and reinforced AESO foams are shown in Figure 1. It can be seen from the SEM images that the pores are randomly oriented with pore shapes that are semi-spherical and mostly highly irregular, from the side view of the materials. Nevertheless, the pore size is highly non-uniform throughout the samples.

**Figure 2:**
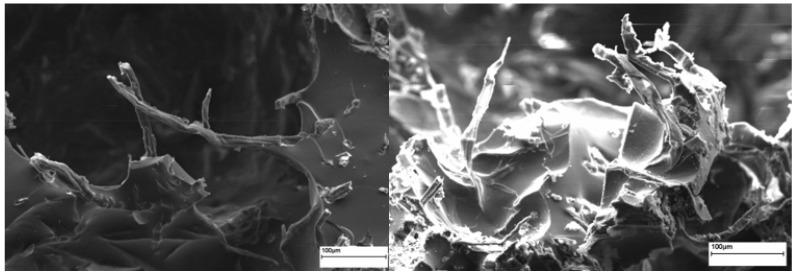
The SEM images of cellulose fibres in AESO 4 foams.

Foam is the dispersion of a gas in a liquid, which creates a characteristic structure when the matrix solidifies. Foams are usually prepared with liquid matrices. Because of the large density difference between air and liquid, the employed liquid has a tendency to drain. The stability of liquid foams is governed by the dynamics of the thin interfacial films formed between air bubbles approaching each other. Two main reasons for foam destabilisation are gravity and/or capillary drainage, which induce film thinning and possible film rupturing unless prevented by repulsive electrostatic or steric forces between the film surfaces [19]. The foam that did not contain cellulose fibres exhibited rapid destabilisation due to the expected fast kinetics of gravitational drainage, followed by capillary drainage, which ultimately resulted in the full phase separation of the foam. When the monomer phase contained cellulose fibres, the kinetics of destabilisation were significantly reduced (Figure 2). It has been proposed that cellulose fibres in the liquid phase will aggregate in the Plateau border, obstructing the flow of the liquid from the foam film [1].

**Figure 3:**
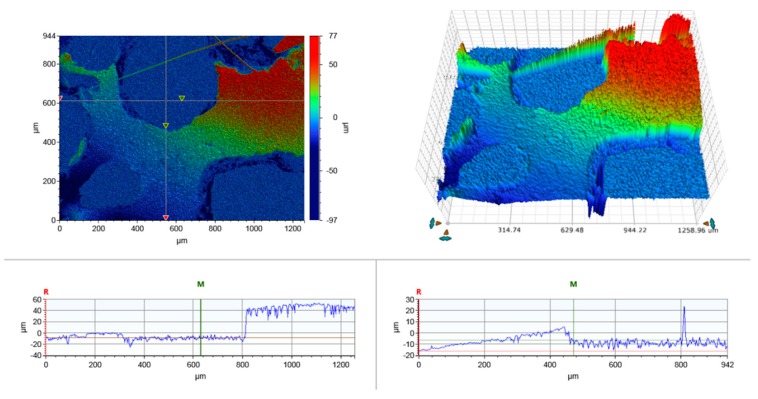
The 3D images of AESO foam reinforced with 2% cellulose fibres.

It can be seen in Figure 1b–d that the addition of cellulose fibres resulted in an increase of cell size likely due to the increased number of nucleating sites induced by the cellulose fibre surfaces [20]. At the same time, the cellulose fibre-reinforced foams had a less uniform pore structure, which should be related to the cellulose fibre distribution within the polymer matrix and the fibre-matrix interactions. It is suggested that, as a result of local fibre-matrix debonding, holes are induced where the gas loss hinders the cell growing ability, and thus the non-uniform distribution of cell size is obtained (Figure 3). With increasing the cellulose fibre loading, the monomer phase became more viscous and the expansion of the gas bubbles in the monomer phase was hindered. This state of the bubbles resulted in the randomly orientated pores.

Porosity is one of the important parameters that govern the physical properties of polymer foams. Grain volume, bulk volume, grain density, and bulk density, along with the calculated porosity (Equation (1)) of cellulose-reinforced AESO foams, are tabulated in Table 1. It can be seen that the porosity increased from 4.5% to 57.0% after incorporating cellulose fibres. The introduction of cellulose fibres into the monomer phase resulted in a stabilisation of the gas-soybean oil interface during thermal polymerisation, and thus in rise of total porosity. The highly porous nature of the cellulose-reinforced AESO 3 foam is a direct result of the mixing process to introduce air bubbles into the bio-based soybean oil resin. It can also be seen from Figure 1c that the porous polymer had the largest cell size compared to AESO 2 and AESO 4 foams. The larger pore size observed in AESO 3 is due to the phase separation of the liquid bio-based foam, as liquid foams are inherently unstable [5].

The density of solid polymeric foams typically range from 0.016 g/cm^3^ to 0.960 g/cm^3^, according to the requirements of a broad range of applications. High-density foams usually have high mechanical strength and are used as lightweight structural components for furniture, construction, and transportation. Medium-density foams are mostly used in the packaging industry, but with satisfactory rigidity they can also be utilised in the automotive industry. Foams produced in this study are on the border between medium- and high-density materials. Furthermore, for structural applications, a strong resistance to deformation is desired, and medium- to high-density foams are preferred [18].

### 3.2. Mechanical Properties of Bio-Based Foams

The compressive strength of porous materials is an important parameter that determines the application of foam. The compressive strength of the pristine AESO material and the cellulose fibre-reinforced AESO foams is shown in Table 2. It was observed that the compression strength of the fibre-reinforced foam increased with increasing cellulose content. This general pattern of an increase in strength can be attributed to the increased load-bearing capacity of the fibrous reinforcements. Thus, the results indicated that the introduction of fibres assisted in altering the response of the material during compressive loading. When the foam cells are completely closed, the foams behave like solids, which corresponds to a gradual rise in compressive stress. Therefore, it is clear that the pristine AESO material exhibited a compressive strength five times higher than that of the cellulose fibre-reinforced foams.

**Figure 4:**
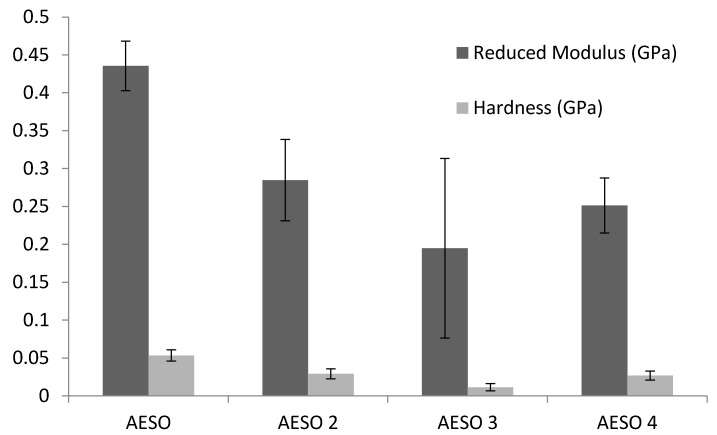
The indentation hardness and reduced modulus for pristine AESO and cellulose-reinforced AESO foams.

The mechanical properties of the foams based on AESO and AESO reinforced with cellulose fibres were also studied by the nanoindentation method. Nanoindentation can provide useful information on the cell wall properties such as nanoindentation reduced modulus and hardness. The values of the nanoindentation reduced modulus and hardness of the cured AESO foams were calculated with Equations (2) and (3), respectively, and are shown in Figure 4. It can be found that the indentation hardness of the foams based on AESO was higher compared to that of AESO reinforced with cellulose fibres; the same trend is observed for the reduced modulus.

It is known from the literature that polymer AESO will have higher local hardness values than AESO foam [21]. This increase in hardness value can be explained due to indenting compact AESO material. Regarding the load-displacement curves for the pristine AESO foams, all of them presented similar penetration depths and slopes, indicating that the AESO foam maintained its properties from the inner to the outer regions.

Porous materials show several types of heterogeneity at the microscale. The heterogeneity can originate from unreacted components or from chemical reactions that are evolving after the mixing. AESO foam is a material with both these heterogeneities, and the material properties of the foam cell walls are also affected by adding stabilising and foaming agents to the mixture during the manufacturing process. The overall behaviour of AESO foam is directly dependent on the formation of the individual phases and their micro-properties. With decreasing pore sizes and pore distances, the influence of the interphase material around the pore becomes more important, and characteristic changes in the nano- and micro-deformation mechanisms appear [22]. Under load, structural openings, variations of local composition or orientation, nano-sized pores degrade the mechanical properties of the material.

The effect of the cellulose fibre content on the mechanical properties of the material can be described based on experimentally determined results. Increasing the cellulose fibre content from 2.0% to 3.0% led to a decrease in the hardness and a reduced modulus. Contrarily, increasing the cellulose fibre content from 3.0% to 4.0% in the matrix caused an increase in the hardness and reduced the modulus. However, the specific data were dependent on the specimen surface. The degradation of the mechanical properties of AESO 3 can be linked to the highest porosity and pore size of the AESO 3 samples, compared to those of AESO 2 and AESO 4. The investigations indicated that the mechanical properties can be enhanced by the higher cellulose fibre content. However, the hardness value and reduced modulus of cellulose-reinforced AESO foams are similar to the values for pure polyurethane foam used in structural applications such as the automobile and wind mill blade industries [23].

### 3.3. Thermal Behaviour of Bio-Based Foams

**Figure 5:**
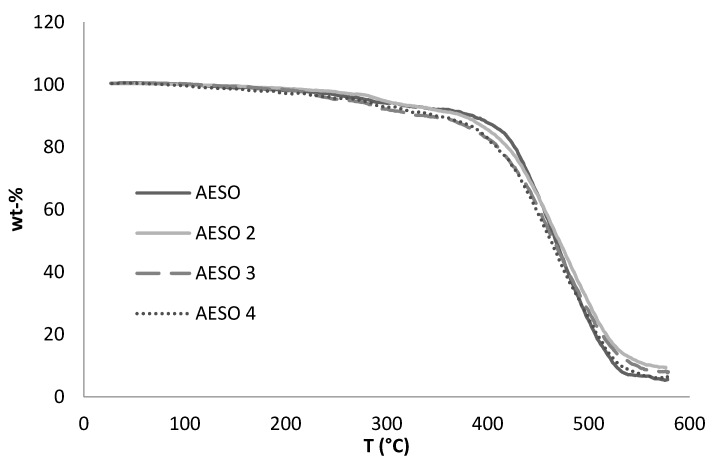
The thermal behaviour of pristine AESO and cellulose-reinforced AESO foams.

The thermal degradation behaviour of the pristine AESO material and cellulose-reinforced AESO foams is shown in Figure 5. It can be seen that all the samples undergo single step degradation in argon atmosphere. Random polymer chain cleavage occurred during the degradation of acrylated epoxidized soybean oil [24]. The incorporation of cellulose fibres into the specimen did not alter the degradation behaviour of the final bio-based foam. This might be due to the low cellulose fibre content in the sample. The onset degradation temperature determined from TGA analysis of the foams was found to be 380 °C for all samples. The residual carbon content was found to be slightly higher for samples containing cellulose fibres compared to the pure soybean oil polymer. The increase in the residual carbon content can be explained by the carbonisation of cellulose fibres in the mixture. The TGA thermogram clearly indicates the good thermal stability of the cured foams up to 300 °C with a minimum amount of weight loss, which may be due to the minimum amount of unreacted components.

## 4. Conclusions

Bio-based foams from cellulose fibres and an acrylated epoxidized resin were produced in this study. The resin was mixed with different concentrations of cellulose fibre and additives, and was thermally polymerised. The results show that it is possible to produce cellulose-reinforced AESO foams with satisfactory mechanical properties without adding a reactive comonomer, such as styrene, to the resin. The resulting cellulose-reinforced AESO foams possessed a porosity of approximately 56% and were in the range of medium-density foams. It was found that the stability of the gas-AESO interface was poor in comparison to the gas-AESO interface containing cellulose fibres. Both reduced modulus and hardness show that with increasing the cellulose fibre content up to a certain amount, desired mechanical properties can be observed. The incorporation of cellulose fibres into the polymeric foams exhibited higher values of the mechanical properties. The compression test results indicated that introduction of fibres assisted in altering the response of the foams during compressive loading. This was attributed to the reinforcing effect of the cellulose fibres. The thermal behaviour of cellulose-reinforced AESO foams was not affected by the addition of cellulose fibres. SEM images showed randomly oriented pores with irregular shapes and non-uniform pore size throughout the samples.

## Figures and Tables

**Table 1:** The porosity, volumes and density of the pristine AESO and cellulose-reinforced AESO foams.

Samples	Porosity *ɛ* (%)	Grain Volume *V*_G_, (cm^3^)	Bulk Volume *V*_B_, (cm^3^)	Grain Density (g/cm^3^)	Bulk Density (g/cm^3^)
AESO	4.5 ± 4.4	1.58 ± 0.05	1.65 ± 0.06	1.55 ± 0.04	1.48 ± 0.04
AESO 2	57.0 ± 1.8	1.92 ± 0.05	4.45 ± 0.15	1.28 ± 0.02	0.538 ± 0.004
AESO 3	58.3 ± 1.5	2.16 ± 0.05	5.17 ± 0.15	1.134 ± 0.013	0.474 ± 0.003
AESO 4	54.2 ± 1.8	2.08 ± 0.05	4.55 ± 0.15	1.177 ± 0.014	0.538 ± 0.004

**Table 2:** Mechanical properties of the pristine AESO and cellulose-reinforced AESO foams.

Samples	Maximum Load (N)	Yield Strength (MPa)	Compressive Strength (MPa)
AESO	1498.0 ± 157.6	28.4 ± 3.3	29.9 ± 3.1
AESO 2	351.2 ± 11.5	3.7 ± 0.2	4.7 ± 0.1
AESO 3	558.5 ± 11.0	4.3 ± 0.2	5.5 ± 0.1
AESO 4	589.9 ± 16.7	4.0 ± 0.2	5.9 ± 0.1
